# Inequalities in Life Expectancy by Education and Its Changes in Lithuania during 2001–2014

**DOI:** 10.3390/medicina57030245

**Published:** 2021-03-05

**Authors:** Olga Mesceriakova-Veliuliene, Ramune Kalediene, Skirmante Sauliune, Gvidas Urbonas

**Affiliations:** 1 Department of Health Management, Faculty of Public Health, Lithuanian University of Health Sciences, Tilžės St. 18, LT-47181 Kaunas, Lithuania; ramune.kalediene@lsmuni.lt (R.K.); skirmante.sauliune@lsmuni.lt (S.S.); 2 Department of Bioethics, Faculty of Public Health, Lithuanian University of Health Sciences, Tilžės St. 18, LT-47181 Kaunas, Lithuania; gvidas.urbonas@lsmuni.lt

**Keywords:** life expectancy, education, inequalities, Lithuania

## Abstract

*Background and Objectives*: Reduction of health inequalities is a highly important task in public health policies worldwide. In Lithuania, inequalities in life expectancy (LE) by education level are among the greatest, compared to other European countries. However, studies on inequalities in LE by level of education over a long-term period are quite scarce in Lithuania. The aim of the study was to analyze inequalities in life expectancy by education and its changes in Lithuania during 2001–2014. *Materials and Methods:* Information on deaths (in population aged ≥30 years) was obtained from Statistics Lithuania. Life expectancy at age 30 (LE30) and 95% confidence intervals (CIs) were calculated using life tables. Inequalities in LE30 were assessed using rate differences. Joinpoint regression analysis was used to assess the trends and inequalities of LE30 during 2001–2014. *Results:* During 2001–2014, LE30 in males and females with post-secondary education was higher than in those with up-to-secondary education (*p* < 0.05). Among males and females, LE30 increased in both education groups, except for males with up-to-secondary education. Among individuals with post-secondary education, LE30 started increasing earlier and more quickly than in those with up-to-secondary education. Over the analyzed period, greater differences in LE30 between post-secondary and up-to-secondary education groups were found among males. Differences in LE30 due to different educational background were statistically significantly, increasing across the sexes with a more rapid increase for females than for males. During 2001 and 2014, the highest number of years of LE30 lost in both education groups was due to cardiovascular diseases. *Conclusions:* Throughout the period of 2001–2014, life expectancy in Lithuania in the post-secondary education group was statistically significantly longer and was increasing more rapidly compared to the up-to secondary education group. Inequalities in life expectancy by level of education significantly increased among both males and females.

## 1. Introduction

The health of the population is a national capital, and health promotion should be one of the primary goals of each state [1]. Investments in public health are investments in economic growth and prosperity. Therefore, most countries are aiming at reducing health inequalities, increasing life expectancy (LE), and improving the population’s health. Health inequalities are a persistent problem in European countries and one of the main challenges for public health [2]. Health inequalities are caused by adverse socio-economic and environmental circumstances [3]. Reduction of socio-economic inequalities in health is one of the most important goals of health policy in the European Union (EU) countries [1,4,5,6].

Lithuania, along with other countries, pays much attention to the reduction of health inequalities. Following the Health 2020, the European policy framework and strategy for the 21st century published by the World Health Organization (WHO), and good practice of the EU member states, the Ministry of Health of the Republic of Lithuania approved the Lithuanian Health Strategy 2014–2025 and the Action Plan for Reducing Health Inequalities [1,4]. These documents aim at improving the health of Lithuanian population, reducing mortality rates, increasing LE, reducing health inequalities, and improving access to health care across regions and social groups. LE is one of the main indicators for the surveillance of inequalities in health [7]. Large inequalities in LE exist depending not only on sex, but also socio-economic status, irrespective of whether it is measured by education level, income, or occupational group [8,9,10,11,12]. However, LE by educational attainment is the most important indicator of socio-economic inequalities in health [8,12]. Inequalities in LE between the poorly and better educated populations prevail in all European countries [11,12,13]. LE is shorter among persons with the lowest educational attainment and increases with improving educational levels [13]. Education level influences health by affecting lifestyle-related risk factors such as smoking, alcohol consumption, dietary patterns, and physical inactivity, as well as by its impact on financial resources, housing and working conditions, and access to care [14].

Inequalities in LE in general and in LE compared by education level are observed as larger among males than females, especially in Central and Eastern Europe [11,13]. During the last two decades, the LE of Lithuanian population increased by more than four years—from 72.1 years in 2000 to 76.4 years in 2019. However, the increase in LE of the Lithuanian population is significantly slower and is one of the smallest among the European Union countries [15].

Commonly, larger health inequalities are observed in countries undergoing intense social, economic, and political changes [16], including Lithuania, with the largest LE inequalities among Eastern European countries. Nevertheless, standards of living, economic situation, quality and accessibility of health care services, and health literacy of the population have been increasing in Lithuania [17], including educational level.

According to the Constitution of Lithuania, education in Lithuania is mandatory for children aged 6/7 to 16 and is free of charge at all educational levels [18]. In 2011, there were 212 persons with higher education (per 1000 population), 1.7 times higher as compared to 2001 (126 persons per 1000 population). In addition, the proportion of the population with primary education decreased by one-third over the decade. Compared by gender, a higher proportion of females obtained higher education as compared to males—23.7 and 18.3 per cent, respectively (in comparison, 13.5 and 11.5 per cent, respectively, in 2001) [19].

Due to significant health inequalities observed in Lithuania, a decision was made to analyze inequalities in LE by education level over a 14-year period. The period of 2001–2014 was chosen due to data availability. This is the first analysis of this type in Lithuania. The aim of this study was to evaluate changes in LE inequalities by education level in Lithuania during 2001–2014.

## 2. Materials and Methods

### 2.1. Data Sources

A record-linked cohort study covering the entire Lithuanian population was conducted. The routine data were used from Statistics Lithuania (2001 and 2011 population censuses—individual records containing information on sex, date of birth, and education level) and the National Mortality Register (data on individual causes of death using the coding of the International Classification of Diseases (ICD)-10, sex, and dates of birth and death covering the periods from 6 April 2001 to 31 December 2014), while the Population Register (individual data on declared emigration from Lithuania during the period from 6 April 2001 to 31 December 2014) was used for a more precise assessment of the population size.

Individual records from these databases were linked using personal identification numbers by the staff at Statistics Lithuania who have access and the necessary permission to handle data containing personal identification numbers. After the linkage was performed, the identification numbers were removed, and the resulting anonymized data set was used for further analysis. The linked data set contained the records of individuals who were 30 years old or older at the time of population censuses on 6 April 2001 and on 1 March 2011. The study included only the population aged 30 years or older because it was likely that the younger age groups had not yet completed their education, and thus the data on such groups would not be reliable.

### 2.2. Population

Records covering the entire 14-year period were further split into yearly intervals, allowing for the calculation of annual LE for trends assessment. The total number of such yearly records reached 24,851,707, and this file was the basis for most subsequent analyses. The total volume of follow-up data was 25,346,450 person-years and 525,522 cases of death. Of all the registered deaths in the mortality register data, 6% were unlinked—i.e., no corresponding personal ID numbers were found in the population census database. These cases were excluded from the analysis.

More detailed information on person-years and the number of deaths for each educational attainment group for each year is presented in the Supplementary Material (see Table S1).

### 2.3. Statistical Analysis

The life tables by sex and the level of education (up-to-secondary (unknown, preprimary or no education, primary, lower secondary, or upper secondary) and post-secondary (post-secondary non-tertiary or tertiary)) were calculated, and 95% CIs of LE30 were estimated. We calculated the number of years of life lost in LE30 due to the four major causes of death in Lithuania—cardiovascular diseases (ICD-10 codes I00-I99), cancer (ICD-10 codes C00-C97), external causes (ICD-10 codes V01-Y98), and diseases of the digestive system (ICD-10 codes K00-K93). More detailed information on the number of deaths from major causes by the level of education over the period of 2001–2014 is presented in the Supplementary Material (see Tables S2–S5).

Changes in the magnitude of LE30 inequalities by level of education were assessed using the easily interpretable measures of absolute inequalities (rate difference (RD) = post-secondary − up-to-secondary). For the assessment of inequality trends and LE30 by level of education during 2001–2014, joinpoint regression analysis was applied. Joinpoint regression is a Windows-based statistical software program that enables a user to test the statistical significance of an apparent change in a trend. In this analysis, the best-fitting points, where the rate changes increased or decreased significantly, were chosen [20]. The analysis started with a minimum number of cut points, testing whether one or more cut points were statistically significant and whether they could be added to the model. In the final model, each joinpoint indicated a statistically significant change in a trend; computed next was the annual percent of change for each of those trends. For the joinpoint analysis, the overall significance level was set at *p* = 0.05. Significant changes included changes in the direction or rate of the trend. The permutation test—testing the number of cut points 0 against 1—was applied in this case because the 14-year period did not allow for obtaining statistically significant results for more joinpoints. Coefficients of regression multiplied by 100 were presented as average annual changes, which were statistically significant at *p* < 0.05.

## 3. Results

**Figure 1:**
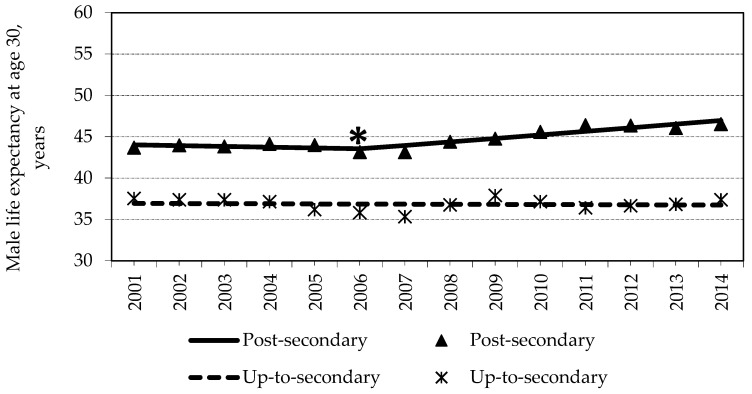
Life expectancy of males and its changes in education groups during 2001–2014. * cut point is statistically significant, *p* < 0.05.

Among males with post-secondary education, LE30 was observed to be longer by 2.86 years in 2014 as compared to 2001 (*p* < 0.05) (Figure 1). The joinpoint regression analysis revealed that the increase in LE30 was not gradual within this group: a statistically significant cut point was detected in 2006. During 2001–2006, LE30 decreased annually by 0.21% per year, on the average, from 43.69 (95% CI = 43.24–44.13) years in 2001 to 4 3.16 (95% CI = 42.78–43.54) years in 2006 (*p* = 0.543). Thereafter, LE30 started to increase annually by 0.95%, on the average, from 43.16 (95% CI = 42.78–43.54) in 2006 to 46.55 (95% CI = 46.23–46.87) in 2014 (*p* = 0.0002). Among males with up-to-secondary education, negative, albeit statistically non-significant, trends were observed: LE30 was shorter by 0.16 years in 2014 compared to 2001 (*p* > 0.05). More detailed information on LE30 with confidence intervals for each educational group for each year is presented in the Supplementary Material (see Table S6).

**Figure 2:**
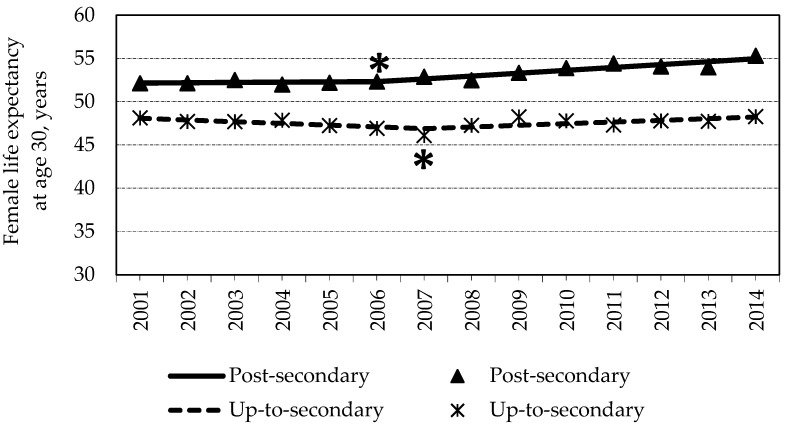
Life expectancy of females and its changes via education groups during 2001–2014. * cut point is statistically significant, *p* < 0.05.

LE30 of females with post-secondary education increased more rapidly than that in the up-to secondary education group during 2001–2014 (Figure 2). In 2014, LE30 for females with post-secondary educational was statistically significantly longer (by 3.19 years) as compared to 2001. The joinpoint regression analysis revealed that LE30 of females with post-secondary education increased inconsistently throughout the study period—a statistically significant cut point was found in 2006. During 2001–2006, LE30 increased by 0.06% per year, on average, from 52.13 (95% CI = 51.68–52.58) in 2001 to 52.33 (95% CI = 52–52.66) in 2006, and the change was statistically non-significant (*p* = 0.810). Thereafter, during 2006–2014, LE30 increased by 0.62% annually, on the average, from 52.33 (95% CI = 52–52.66) in 2006 to 55.32 (95% CI = 55.01–55.63) in 2014 (*p* = 0.0003).

Meanwhile, LE30 among females with up-to-secondary education was longer only by 0.16 years in 2014 as compared to 2001 (*p* > 0.05). This happened because LE30 decreased by 0.42% per year, on average, from 48.12 (95% CI = 47.85–48.4) in 2001 to 46.1 (95% CI = 45.82–46.38) in 2007 (*p* = 0.039). Starting from this statistically significant cut point, LE30 increased by 0.4% per year, on average, from 46.1 (95% CI = 45.82–46.38) in 2007 to 48.28 (95% CI = 47.96–48.59) in 2014 (*p* = 0.016). More detailed information on LE30 for each educational attainment group for each year is presented in the Supplementary Material (see Table S6).

**Figure 3:**
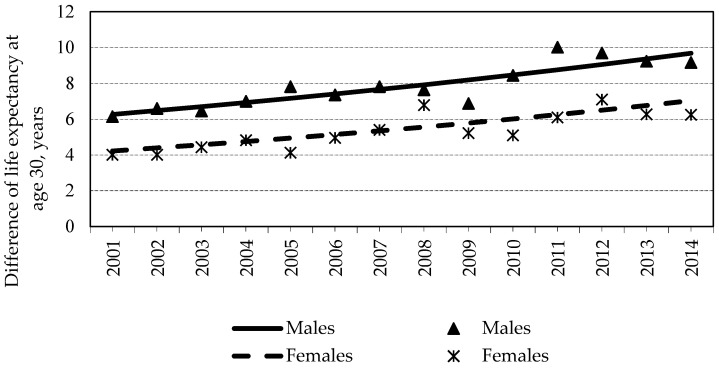
Trends in differences of life expectancy by level of education and sex during 2001–2014.

For the assessment of trends in the differences of LE30 by education and sex during 2001–2014, the joinpoint regression analysis was performed. A statistically significant increase in differences by the level of education was detected (Figure 3). However, no statistically significant cut points were found. More pronounced and statistically significant differences between different educational attainment groups were observed among males that among females. However, inequalities in LE30 between different educational groups in females increased more rapidly (from 4.01 (95% CI = 3.83–4.18) in 2001 to 7.04 (95% CI = 6.73–7.04) in 2014—on the average, by 4% per year (*p* = 0.0001)) as compared to males (from 6.15 (95% CI = 5.99–6.3) in 2001 to 9.17 (95% CI = 9.17–9.18) in 2014—on average, by 3.4% per year (*p* < 0.0001)). More detailed information on differences of LE30 for each educational group for each year and CI is presented in the Supplementary Material (see Table S6).

Years of LE30 lost due to the main causes of death among males and females by education level in 2001 and 2014 are presented in Table 1. In 2001 and 2014, males and females in the up-to secondary education group lost more years of potential life due to cardiovascular diseases, external causes, and diseases of the digestive system compared to those in the post-secondary education group. However, those in the post-secondary education group lost more years of potential life due to cancer. In the up-to secondary education group, males lost more years of life compared to females, while in the post-secondary education group, females lost more years of life than males. In 2014, compared to 2001, the number of years of LE30 lost due to external causes or cardiovascular diseases decreased (with an exception for females in the up-to secondary education group).

In both education groups, the highest number of years of LE30 lost was due to death from cardiovascular diseases. However, the importance of these causes was more significant for females than for males. In males, the lowest number of years of LE30 lost was due to deaths from diseases of the digestive system while, in females, due to diseases of the digestive system in 2001 and external causes in 2014. The analysis of differences in the number of years of LE30 lost due to the main causes of death by education level in 2001 and 2014 revealed more differences for males and for females in the year 2014 compared to 2001. By education level, both in 2001 and 2014, the greatest differences in the number of years of LE30 lost was due to deaths from external causes for males and due to deaths from cardiovascular diseases for females.

## 4. Discussion

Although studies of inequalities in LE based on sex and differences in education level have been performed in Lithuania before, inequalities in LE were analyzed over a shorter time period and in different educational and age groups [21,22,23,24]. This is one of the largest studies conducted in Lithuania, which linked the causes of death in the Lithuanian population to the data of two population censuses and records in the population register for the period of 2001–2014. In this study, the LE in the up-to secondary and post-secondary education groups was evaluated among males and females, and changes in LE inequalities by the level of education were evaluated for the first time in Lithuania and for such a long period. One of the greatest advantages of this study is the precise information on the socio-economic status of deceased population groups, as data were collected during the population census rather than reported by family members [25,26] Another advantage of this study is the choice of a lower age limit of 30 years. This age limit was chosen purposefully because, after this age, the education of the population changes little. However, the important limitation of such studies is that the socio-economic status of the population can change after the census. Nevertheless, our results show that attainment of post-secondary education until the age of 30 might be a factor that improves life expectancy among males and females. In terms of practical applicability for routine monitoring of inequalities, the education variable seems to be most suitable due to the simplicity of measurement and stability over time after the main education is completed.

Our study showed that at the age of 30+ years, LE of males and females in the post-secondary education group was longer than that in the up-to secondary education group throughout the study period. Eurostat data also suggest that in most European countries, similar trends are predominant with the exceptions of the Czech Republic, Greece, Croatia, Italy, and Romania. In these countries, LE at the age of 30+ years was longer among females in the less educated groups, albeit not during all periods—e.g., in 2014 in Croatia, LE for females with a low level of education was by as many as 2.4 years longer than that for females with a high level of education [13].

One of the aims of the First Lithuanian Health Program was to increase LE of the Lithuanian male and female population, and this goal was successfully achieved [27]. However, according to our data, LE trends among males and females at age 30 by level of education were not equally favorable in all education groups and not during the whole study period. It was estimated that LE at age 30 was increasing only among males and females in the post-secondary education group and among females in the up-to-secondary education group. LE among the population in the post-secondary education group started to increase significantly earlier and was increasing more rapidly compared to that in the up-to-secondary education group.

2006–2007 years were identified as the joinpoints signifying the end of negative and the beginning of positive trends in LE30. It is interesting to note that this finding is complementary to the national statistical data showing increase in mortality from the major causes of death (cardiovascular diseases, cancer, and external causes) in 2006–2007 [28]. Other studies found that the highest mortality rates were dominant among the most vulnerable groups (widowed, lower educated [29], and rural males [28]). Other studies linked increase in mortality with alcohol consumption [30]. Previously liberal, and now strict alcohol control measures were implemented in Lithuania in 2006, and that might explain the change in LE30 trends, especially for people with post-secondary education. However, the link is to be examined in future studies.

In other EU countries, in contrast to Lithuania, LE increased among males and females with low education, except in Slovakia among females. In the population with tertiary education, there was even a declining trend in LE, especially among females (in Bulgaria, the Czech Republic, Greece, Italy, Hungary, and Romania) [13]. LE decreased the most in the Czech Republic—in 2017, it was by as many as 4 years shorter than that in 2007 (in the age group of 30+ years).

In Lithuania, these favorable trends can be related to an increasing awareness of the population—those in the post-secondary education group more often apply their knowledge, information, and experiences in health promotion and disease prevention behaviors, and thus increase their LE [31,32]. In addition, declining mortality rates in these education groups [33], declining numbers of deaths at a young age (especially alcohol-related deaths) [28], declining infant and maternal mortality [28], increasing healthy LE [13], increasing quality and accessibility of health care services [34,35], and economic growth [15] are important factors for the explanation of such trends. One of the implications following from the results of the study is the need to improve health literacy at the early stages of education and promote healthy lifestyle habits to reduce the gap in LE between the educational groups.

Despite significant efforts in reducing health inequalities in Lithuania and in most EU countries, inequalities in LE by level of education remain [13]. According to our findings and data published by other authors, differences between education groups are most pronounced among males [11,13,21]—especially in Central and Eastern European countries (Slovakia, Poland, Hungary, and the Czech Republic), where the LE gap between males with higher and lower education levels was more than ten years in 2017 [13]. Despite the lack of data on LE by level of education in Lithuania in 2017, other authors found greater than 10-year differences in LE between different education groups among males [21,22,23,24]. According to our data, such significant differences were observed only in 2011. This can be largely explained by the fact that the older population in Lithuania and in other Central and Eastern European countries frequently has lower levels of education, higher mortality rates, and a greater prevalence of risk factors, such as tobacco and/or alcohol use among males [11]. Due to different LE at age 30 and different trends in up-to-secondary and post-secondary education groups, differences between education groups among males and females were increasing statistically significantly throughout the study period. However, among females, differences in LE were increasing more rapidly, which may be related to a more rapid increase in LE in females in the post-secondary education group. Increasing inequalities in LE have also been observed in other countries of the EU. Among males, inequalities in LE are increasing in Sweden, Portugal, and Slovakia, while among females, they are increasing in Denmark, Slovenia, Slovakia, and Finland [13]. In other countries, differences in LE are decreasing. The most rapid decrease in LE differences was observed in Estonia: in 2016, the differences in LE among males and females were smaller by, respectively, 11.1 and 4.3 years than in 2007, yet the differences remained among the largest. However, the results of our study indicate that not all aims related to the reduction of health and social inequalities listed in the first Lithuanian Health Program, the EU, and the WHO political documents have been implemented in Lithuania [27,36]. In most EU countries, the reduction of health inequalities among different socio-economic groups has already been one of the most important directions in health policies for several decades [36]. Meanwhile, in Lithuania, considerable efforts in reducing inequalities in health were initiated only in 2014 [1,4]. Therefore, it can be expected that the implementation of the Lithuanian Health Strategy and the Action Plan for Reducing Health Inequalities will help to reduce growing health inequalities.

## 5. Conclusions

The study showed that at age 30, LE of Lithuanian males and females in the post-secondary education group was statistically significantly longer and was increasing more rapidly compared to that in the up-to secondary education group during 2001–2014. Inequalities in LE30 by education levels were increasing due to different trends in the increase of LE30 throughout the study period. Inequalities in LE30 by education level were more significant among males, while the increase in inequalities was significantly more rapid among females (on average, by 4% per year, *p* = 0.0001) than among males (on average, by 3.4% per year, *p* < 0.0001). In 2001 and 2014, the deceased in the up-to secondary education group lost more years of LE30 due to cardiovascular diseases, external causes, and diseases of the digestive system compared to those in the post-secondary education group. The highest number of years of LE30 lost in both education groups was due to cardiovascular diseases in both 2001 and 2014.

## Figures and Tables

**Table 1:** Years of life expectancy lost due to the main causes of death by education level in 2001 and 2014.

Causes of Death	Sex	2001			2014		
		Up-to- Secondary	Post- Secondary	Difference *	Up-to- Secondary	Post- Secondary	Difference *
Cardiovascular diseases	Males	7.39	6.93	0.46	6.91	6.50	0.41
	Females	7.85	6.13	1.72	9.78	5.56	4.22
Cancer	Males	2.95	3.31	−0.36	3.07	3.58	−0.51
	Females	2.56	3.36	0.80	2.83	4.08	−1.25
External causes	Males	3.67	2.68	0.99	3.13	1.35	1.78
	Females	1.21	0.74	0.47	0.97	0.62	0.35
Diseases of the digestive system	Males	0.66	0.45	0.21	1.09	0.68	0.41
	Females	0.60	0.36	0.24	1.03	0.70	0.33

* differences between the up-to-secondary and the post-secondary education groups.

## Data Availability

Data can be made available from the corresponding author on reasonable request.

## Supplementary Materials

The following are available online at https://www.mdpi.com/1648-9144/57/3/245/s1, Table S1: distribution of person-years and deaths by level of education during 2001–2014, Table S2: distribution of deaths from cardiovascular diseases by level of education during 2001–2014, Table S3: distribution of deaths from cancer by level of education during 2001–2014, Table S4: distribution of deaths from external causes by level of education during 2001–2014, Table S5: distribution of deaths from diseases of the digestive system by level of education during 2001–2014. Table S6: life expectancy and differences with confidence intervals (CI) by level of education during 2001–2014.
