# Supplementary material for: Inequalities in Life Expectancy by Education and Its Changes in Lithuania during 2001–2014
Source: Medicina (Kaunas). 2021 Mar 5;57(3):245. doi: 10.3390/medicina57030245 (PMC8001735; doi:10.3390/medicina57030245)
Supplement: Supplementary file 1 [file medicina-57-00245-s001.pdf]

Supplementary file

Table S1: distribution of person-years and deaths by level of education during 2001-2014.

Years	Males				Females				All	
	Up-to-secondary		Post-secondary		Up-to-secondary		Post-secondary			
	Person-years /%	Deaths /%	Person-years /%	Deaths /%	Person-years /%	Deaths /%	Person-years /%	Deaths /%	Person-years /%	Deaths /%
2001	401640 /26.64	11245 /42.61	260219 /17.26	2640 /10.00	483016 /32.03	10958 /41.52	362911 /24.07	1550 /5.87	1507786 /100	26393 /100
2002	529647 /26.39	15734 /42.13	348749 /17.38	3648 /9.77	639678 /31.87	15745 /42.16	488873 /24.36	2216 /5.94	2006947 /100	37343 /100
2003	513608 /26.10	15595 /41.81	344637 /17.52	3833 /10.28	623435 /31.68	15540 /41.66	485946 /24.70	2335 /6.25	1967626 /100	37303 /100
2004	499037 /25.82	15394 /40.92	340951 /17.64	3947 /10.49	608794 /31.50	15645 /41.59	483702 /25.04	2634 /7.00	1932484 /100	37620 /100
2005	481271 /25.52	16372 /41.27	335370 /17.78	4179 /10.53	590477 /31.31	16415 /41.38	478898 /25.39	2705 /6.82	1886016 /100	39671 /100
2006	464574 /25.19	16336 /40.49	330692 /17.93	4483 /11.11	573554 /31.10	16554 /41.03	475422 /25.78	2970 /7.37	1844242 /100	40343 /100
2007	447801 /24.85	16631 /40.59	325732 /18.08	4831 /11.79	556706 /30.89	16348 /39.90	471836 /26.18	3164 /7.72	1802075 /100	40974 /100
2008	432781 /24.52	15296 /38.72	321509 /18.21	4643 /11.75	541597 /30.68	16152 /40.89	469292 /26.59	3409 /8.64	1765179 /100	39500 /100
2009	416285 /24.21	14474 /38.07	315620 /18.35	4635 /12.19	523666 /30.45	15601 /41.03	463968 /26.99	3314 /8.71	1719539 /100	38024 /100
2010	413212 /24.64	13743 /38.67	307366 /18.33	4198 /11.81	495146 /29.53	14451 /40.66	461156 /27.50	3147 /8.86	1676880 /100	35539 /100
2011	410140 /25.10	13013 /39.37	299112 /18.30	3761 /11.38	466626 /28.55	13301 /40.24	458344 /28.05	2980 /9.01	1634222 /100	33055 /100
2012	475857 /25.1	15233 /38.05	352887 /18.42	4749 /11.86	542991 /28.35	16068 /40.13	543893 /28.39	3985 /9.96	1915628 /100	40035 /100
2013	458074 /24.56	15099 /37.25	346219 /18.56	4953 /12.22	524091 /28.10	16210 /39.99	537016 /28.78	4274 /10.54	1865400 /100	40536 /100
2014	442674 /24.29	14291 /36.47	340595 /18.69	5023 /12.82	507489 /27.85	15601 /39.81	531668 /29.17	4271 /10.90	1822426 /100	39186 /100

Table S2: distribution of deaths from cardiovascular diseases by level of education during 2001-2014.

Years	Males				Females				All	
	Up-to-secondary		Post-secondary		Up-to-secondary		Post-secondary			
	Deaths	%	Deaths	%	Deaths	%	Deaths	%	Deaths	%
2001	5474	37.09	1077	7.30	7594	51.46	613	4.15	14758	100
2002	7906	37.17	1567	7.37	10851	51.02	943	4.44	21267	100
2003	7934	37.39	1623	7.65	10696	50.40	968	4.56	21221	100
2004	7679	35.80	1761	8.21	10871	50.68	1138	5.31	21449	100
2005	8329	36.77	1793	7.91	11374	50.21	1158	5.11	22654	100
2006	8122	35.40	1952	8.51	11533	50.27	1337	5.82	22944	100
2007	8152	35.52	2108	9.18	11286	49.17	1406	6.13	22952	100
2008	7666	34.20	2095	9.35	11163	49.80	1490	6.65	22414	100
2009	7373	33.41	2117	9.59	10994	49.82	1584	7.18	22068	100
2010	6926	33.78	1957	9.55	10054	49.04	1565	7.63	20502	100
2011	6479	34.22	1797	9.49	9113	48.13	1546	8.16	18935	100
2012	7546	32.64	2291	9.91	11198	48.45	2081	9.00	23116	100
2013	7460	32.03	2381	10.22	11220	48.18	2227	9.57	23288	100
2014	6972	31.06	2426	10.81	10767	47.97	2280	10.16	22445	100

Table S3: distribution of deaths from cancer by level of education during 2001-2014.

Years	Males				Females				All	
	Up-to-secondary		Post-secondary		Up-to-secondary		Post-secondary			
	Deaths	%	Deaths	%	Deaths	%	Deaths	%	Deaths	%
2001	2446	45.54	596	11.10	1768	32.92	561	10.44	5371	100
2002	3316	44.26	863	11.52	2531	33.78	782	10.44	7492	100
2003	3214	43.02	894	11.97	2516	33.68	847	11.33	7471	100
2004	3324	43.95	898	11.87	2434	32.18	908	12.00	7564	100
2005	3265	42.73	935	12.24	2551	33.39	890	11.64	7641	100
2006	3293	42.72	1038	13.46	2466	31.99	912	11.83	7709	100
2007	3418	43.51	1085	13.81	2373	30.21	980	12.47	7856	100
2008	3174	40.44	1111	14.16	2455	31.28	1108	14.12	7848	100
2009	3228	41.70	1132	14.62	2343	30.27	1038	13.41	7741	100
2010	2989	41.44	1051	14.57	2199	30.49	974	13.50	7213	100
2011	2749	41.13	970	14.51	2055	30.75	910	13.61	6684	100
2012	3203	40.34	1234	15.54	2380	29.97	1123	14.15	7940	100
2013	3130	40.06	1176	15.05	2308	29.54	1200	15.35	7814	100
2014	3131	39.29	1361	17.08	2270	28.49	1207	15.14	7969	100

Table S4: distribution of deaths from external causes by level of education during 2001-2014.

Years	Males				Females				All	
	Up-to-secondary		Post-secondary		Up-to-secondary		Post-secondary			
	Deaths	%	Deaths	%	Deaths	%	Deaths	%	Deaths	%
2001	1708	56.54	622	20.59	515	17.05	176	5.82	3021	100
2002	2156	57.07	718	19.00	693	18.34	211	5.59	3778	100
2003	2072	55.42	762	20.38	696	18.61	209	5.59	3739	100
2004	2046	56.26	691	19.00	679	18.67	221	6.07	3637	100
2005	2169	55.43	786	20.09	698	17.84	260	6.64	3913	100
2006	2098	55.77	724	19.25	664	17.65	276	7.33	3762	100
2007	1976	55.07	723	20.15	675	18.81	214	5.97	3588	100
2008	1711	52.66	672	20.68	623	19.18	243	7.48	3249	100
2009	1474	52.87	638	22.88	471	16.89	205	7.36	2788	100
2010	1523	56.10	527	19.41	480	17.68	185	6.81	2715	100
2011	1572	59.50	416	15.75	489	18.51	165	6.24	2642	100
2012	1796	57.92	514	16.58	575	18.54	216	6.96	3101	100
2013	1733	57.69	553	18.41	514	17.11	204	6.79	3004	100
2014	1607	58.69	463	16.91	493	18.01	175	6.39	2738	100

Table S5: distribution of deaths from diseases of the digestive system by level of education during 2001-2014.

Years	Males				Females				All	
	Up-to-secondary		Post-secondary		Up-to-secondary		Post-secondary			
	Deaths	%	Deaths	%	Deaths	%	Deaths	%	Deaths	%
2001	418	43.36	106	11.00	363	37.66	77	7.98	964	100
2002	586	43.60	152	11.31	521	38.76	85	6.33	1344	100
2003	640	44.29	190	13.15	518	35.85	97	6.71	1445	100
2004	606	40.95	198	13.38	543	36.69	133	8.98	1480	100
2005	715	42.03	245	14.40	604	35.51	137	8.06	1701	100
2006	887	43.12	296	14.39	676	32.86	198	9.63	2057	100
2007	976	41.18	390	16.46	766	32.32	238	10.04	2370	100
2008	889	40.82	312	14.33	736	33.79	241	11.06	2178	100
2009	793	40.44	287	14.64	685	34.93	196	9.99	1961	100
2010	770	42.10	242	13.23	653	35.70	164	8.97	1829	100
2011	747	43.99	197	11.60	622	36.63	132	7.78	1698	100
2012	865	42.44	271	13.30	694	34.05	208	10.21	2038	100
2013	901	41.16	278	12.70	781	35.68	229	10.46	2189	100
2014	836	40.76	266	12.97	742	36.18	207	10.09	2051	100

Table S6: life expectancy and differences with confidence intervals (CI) by level of education during 2001-2014.

Years	Males			Females		
	Up-to-secondary	Post-secondary	Differences (95% CI)	Up-to-secondary	Post-secondary	Differences** (95% CI)
	Life expectancy (95% CI)	Life expectancy (95% CI)		Life expectancy (95% CI)	Life expectancy (95% CI)	
2001	37.54 (37.25; 37.83)	43.69 (43.24; 44.13)*	6.15 (5.99; 6.30)*	48.12 (47.85; 48.40)	52.13 (51.68; 52.58)*	4.01 (3.83; 4.18)*
2002	37.38 (37.13; 37.64)	43.98 (43.61; 44.35)*	6.60 (6.48; 6.71)*	47.70 (47.47; 47.94)	52.14 (51.78; 52.50)*	4.44 (4.31; 4.56)*
2003	37.38 (37.12; 37.65)	43.84 (43.47; 44.20)*	6.46 (6.35; 6.55)*	47.69 (47.44; 47.94)	52.52 (52.18; 52.87)*	4.83 (4.74; 4.93)*
2004	37.15 (36.86; 37.43)	44.15 (43.78; 44.52)*	7.00 (6.92; 7.09)*	47.86 (47.60; 48.11)	51.99 (51.68; 52.31)*	4.13 (4.08; 4.20)*
2005	36.19 (35.87; 36.52)	44.01 (43.59; 44.44)*	7.82 (7.72; 7.92)*	47.24 (46.94; 47.54)	52.20 (51.87; 52.53)*	4.96 (4.93; 4.99)*
2006	35.80 (35.52; 36.09)	43.16 (42.78; 43.54)*	7.36 (7.26; 7.45)*	46.93 (46.68; 47.18)	52.33 (52.00; 52.66)*	5.40 (5.32; 5.48)*
2007	35.33 (35.04; 35.61)	43.15 (42.80; 43.50)*	7.82 (7.76; 7.89)*	46.10 (45.82; 46.38)	52.89 (52.59; 53.19)*	6.79 (6.77; 6.81)*
2008	36.76 (36.49; 37.04)	44.41 (44.07; 44.74)*	7.65 (7.58; 7.70)*	47.27 (47.00; 47.55)	52.49 (52.21; 52.78)*	5.22 (5.21; 5.23)*
2009	37.90 (37.61; 38.19)	44.78 (44.44; 45.12)*	6.88 (6.83; 6.93)*	48.25 (47.97; 48.53)	53.35 (53.07; 53.64)*	5.10 (5.10; 5.11)*
2010	37.16 (36.87; 37.45)	45.61 (45.26; 45.95)*	8.45 (8.39; 8.50)*	47.79 (47.49; 48.08)	53.89 (53.61; 54.17)*	6.10 (6.09; 6.12)*
2011	36.41 (36.13; 36.70)	46.43 (46.08; 46.78)*	10.02 (9.95; 10.08)*	47.32 (47.01; 47.63)	54.42 (54.15; 54.70)*	7.10 (7.07; 7.14)*
2012	36.67 (36.40; 36.95)	46.37 (46.05; 46.69)*	9.70 (9.65; 9.74)*	47.80 (47.52; 48.08)	54.08 (53.83; 54.32)*	6.28 (6.24; 6.31)*
2013	36.84 (36.55; 37.12)	46.08 (45.75; 46.40)*	9.24 (9.20; 9.28)*	47.73 (47.44; 48.02)	53.98 (53.74; 54.22)*	6.25 (6.20; 6.30)*
2014	37.38 (37.06; 37.69)	46.55 (46.23; 46.87)*	9.17 (9.17; 9.18)*	48.28 (47.96; 48.59)	55.32 (55.01; 55.63)*	7.04 (7.04; 7.05)*

*difference is statistically significant; **differences (post-secondary – up-to-secondary).
